# Internet Use in Old Age: Results of a German Population-Representative Survey

**DOI:** 10.2196/15543

**Published:** 2020-11-23

**Authors:** Janine Quittschalle, Janine Stein, Melanie Luppa, Alexander Pabst, Margrit Löbner, Hans-Helmut Koenig, Steffi G Riedel-Heller

**Affiliations:** 1 Institute of Social Medicine, Occupational Health and Public Health (ISAP) University of Germany Leipzig Germany; 2 Department of Health Economics and Health Services Research University Medical Center Hamburg-Eppendorf Hamburg Germany

**Keywords:** internet use, epidemiology, old age, health services, eHealth

## Abstract

**Background:**

The internet has the potential to foster healthy lifestyles and to support chronic disease management. Older adults could benefit from using the internet and other information and communication technology to access health-related information and interventions available online.

**Objective:**

The aim of this study was to investigate factors influencing internet use in older and oldest age groups and to determine the frequency of internet use for health-related purposes.

**Methods:**

Using data from a nationally representative telephone survey of older adults aged 75 years and over, a sample of 999 people was assessed using structured clinical interviews. Descriptive and binary logistic regression analyses were performed.

**Results:**

Overall, 42.6% (418/999) of participants used the internet. Among those, 55.7% (233/417) searched the internet for health-related information. Regression analyses revealed that internet use was significantly associated with younger age (odds ratio [OR] 0.89, 95% CI 0.85-0.92; *P*<.001), male gender (OR 2.84, 95% CI 2.02-4.00; *P*<.001), higher education levels (OR 6.69, 95% CI 4.48-9.99; *P*<.001), a wider social network (OR 1.04, 95% CI 1.01-1.07; *P*=.01), higher health-related quality of life (OR 1.02, 95% CI 1.00-1.03; *P*=.006), lower levels of depressive symptoms (OR 0.89, 95% CI 0.80-0.99; *P*=.04), and higher rates of chronic illness (OR 1.12, 95% CI 1.04-1.21; *P*<.004).

**Conclusions:**

This study provides population-representative data on internet use in old age in Germany. People in the older and oldest age groups participate in online activities. Understanding the factors that are associated with older adults internet use can contribute to developing tailored interventions and eHealth (electronic health) services to improve well-being in older adults.

## Introduction

### Background

Aging is often associated with major changes and stressful life transitions (eg, shrinking social network, relocation, retirement) that have a big impact on well-being, health, and everyday life [1]. Researchers have highlighted the enormous benefits of internet and information and communication technology (ICT) use for the aging population. For instance, these benefits include enhancement of social connectivity [2], prevention or reduction of social isolation [3], increased information about offline leisure and recreational activities [4,5], and increased empowerment through access to information (eg, health-related issues) [6,7]. Various studies have explored factors at the individual level as well as factors of the social context that predict internet use in older age. Results have consistently shown that factors that are positively associated with internet use over 65 are younger age, male gender, higher educational level, married or living with someone else, specifically partners or family members [2,8-12].

However, some researchers have found fault with these findings, arguing that many studies aggregated people of a certain age together into 1 homogenous category [13], or that they did not include older age groups (eg, 70+) [12]. Few studies that focus on older adults (75+) [4,8,12,13] have analyzed sociodemographic correlates, health determinants, and social context correlates of internet use. They did, however, confirm previous results, for example, with regard to age, gender, education, and marital status. Importantly, the authors found that adults aged 75 and older use the internet less frequently than younger seniors, which led them to conclude that the “digital divide” between this age group and other population groups is more pronounced [12,14]. Because of the benefits of digital technologies for older adults, especially for maintaining a healthy lifestyle [15,16], the digital divide by age is worrying. In order to enable future planning of the health care system, reliable information on internet usage behavior and its predictors in the older and oldest age groups is becoming increasingly important. In Germany, apart from continuous surveys on general internet use [17,18] and few studies on internet use for health-related purposes [19,20], there is a lack of research investigating the internet use of the older and oldest German population.

There is a yearly survey conducted by a national television broadcaster using telephone interviews to examine internet use and communication in Germany. The 2018 survey of online behavior was conducted with a sample of 2009 German-speaking persons aged 14 and older [17]. However, this study did not examine the determinants of internet use nor did it focus on older and oldest aged individuals. Therefore, the aim of this study is to investigate the frequency of internet use and factors associated with internet use among the German older and oldest aged 75-99. Based on a German population-representative sample, this study focuses on the following research questions:

- Do older and oldest German adults use the internet?
- Do they search the internet for physical or psychological health information?
- Are there differences between internet users and nonusers in old age?
- What are the correlates of internet use in older and oldest age?

### Conceptual Framework

In order to structure the results of this study, we relied on Anderson’s behavioral model of health service use [21] and applied this conceptual framework to internet usage. The behavioral model [21] contains 3 main underlying factors: (1) “Predisposing variables” which include demographic and relationship characteristics as well as health perceptions; (2) “Need variables” or the direct cause of service use including the amount and type of disease burden or strain (stressors); (3) “Enabling variables” which are both personal/social resources and community-based resources. This assumes that individuals have the resources and knowledge to know about and use appropriate services. These services must also be available.

## Methods

### Study Design and Sample

We analyzed data derived from the project “Needs assessment in the oldest-old: Application, psychometric examination and establishment of the adapted German version of the Camberwell Assessment of Need for the Elderly (CANE),” which was funded by the German Research Foundation (DFG). Within the project, a population-representative telephone survey was carried out on behalf of the University of Leipzig by a leading market, opinion, and social research institute (USUMA GmbH) in Germany between July and October 2016. The sampling method was based on a procedure implemented by the Association of German Market and Social Research Agency (AMD) and included registered and nonregistered telephone numbers of households throughout Germany. During the first step, individuals aged 75 years and older who lived in randomly selected households were identified as a contact person. In the case of more than 1 person aged 75 years and older living in the contacted household, the kish selection grid was applied in order to randomly select the target person within the household. This method is widely applied for selecting members within a household by using a preassigned table of random numbers to determine the target person. The landline telephone sample used to select the prospective households to be interviewed was converted into a representative population sample by weighting it according to key social demographic characteristics (age, gender, regional distribution by federal state). By applying this technique, equal probability of participation for each member of the randomly selected household, and therefore the representativeness of the sample, was ensured [22]. The detailed sample selection process is presented in Multimedia Appendix 1 and is reported in detail by Stein et al [22].

The survey conducted 1193/2823 (42.26%) telephone interviews. Interviews were excluded if dementia was suspected as assessed using the Six-item Cognitive Impairment Test (6-CIT) [23,24]. In the end, 1004 complete interviews with an average duration of 40 minutes were carried out. For the secondary analyses, the following inclusion criteria had to be met: (1) at least 75 years of age and (2) complete data on relevant variables were available. Additionally, 5 individuals were excluded from the study sample due to missing information or incomplete data on internet usage variables, resulting in an analytical sample size of 999 individuals.

### Ethics Approval

The study was approved by the Ethics Committee of the Medical Faculty of the University of Leipzig. Prior to assessment, all study participants provided informed verbal consent.

### Procedure and Instruments

The survey was conducted using standardized structured computer-assisted telephone interviews, which included sociodemographic variables and several standardized instruments. Prior to the survey, the instruments were tested and adapted within a pretest. Furthermore, the interviewers received comprehensive training.

### Dependent Variables

The frequency of internet use was measured using a 5-point scale ranging from 1 to 5 (1=daily, 2=at least once a week, 3=at least once a month, 4=less than once a month, and 5=never). In addition, the frequency of internet searches for physical or psychological health information was assessed by using a 4-point scale ranging from 1 to 4 (1=yes, often; 2=yes, sometimes; 3=yes, but only rarely; and 4=no, never).

### Independent Variables

Based on Anderson’s behavioral model [21], explanatory variables were chosen. The predisposing characteristics include age, gender, marital status (married/with spouse, married/living apart, single, divorced, widowed), education, and living situation (living alone in a private household), living with others (spouse/partner, with other relatives, in a nursing home, assisted living situation, retirement home, other).

The need factors included depressive symptoms, health-related quality of life, number of chronic diseases, loneliness, and experiences of loss. Depressive symptoms were measured using the 15-item short German language version of the Geriatric Depression Scale (GDS) [25]. The GDS is used to identify symptoms of depression in older adults (eg, the basic satisfaction with one’s life). The scale is a self-report instrument that uses a “Yes/No” format. The short version of the GDS showed good psychometric properties. Reliability coefficient estimated by Cronbach α was .81 for the GDS-15. [26]. According to Allen and Annells [27], a cut-off score on the GDS of 4/5 (noncase/case) correspondents to clinically relevant depressive symptoms and was therefore used in this study to screen participants for depression. The number of chronic diseases was measured by using a list of 22 chronic diseases (eg, high blood pressure, heart attack/coronary heart disease, lung disease, stroke, osteoporosis, diabetes, rheumatism, and cancer) with dichotomous response categories (yes/no). The participants were asked whether or not they had been recently diagnosed by a general practitioner with 1 or more of these chronic medical conditions. Health-related quality of life was assessed by using the Visual Analog Scale of the EuroQol 5-Dimensions 5-Level Questionnaire (EQ-5D-5L Questionnaire) or EQ-VAS [28]. The EQ-5D-5L Questionnaire assesses 5 dimensions of health states (mobility, self-care, usual activities, pain/discomfort, and anxiety/depression). With the vertical visual analogue scale (EQ-VAS) the participant is asked to gauge his or her current health state between best and worst imaginable. The EQ-VAS ranges from 0 (worst imaginable health state) to 100 (best imaginable health state). The EQ-VAS is frequently used as a quantitative measure of self-reported health state and has good psychometric properties in late life [29]. Psychometric properties of the German version of the EQ-5D-5L have been evaluated across different diseases [30-33]. Loneliness was assessed using the 3-item short version of the UCLA (University of California, Los Angeles) Loneliness Scale [34]. The scale captures subjective feelings of loneliness and social isolation (eg, the frequency of lack of company). Participants responded on a 3-point scale ranging from 1 (hardly ever) to 3 (often). The α coefficient of reliability is .72, indicating that the 3-item scale is a reliable measure for loneliness in a telephone survey [34]. Experiences of loss were measured using the Leipziger Lebensereignis-Liste (LLL). The LLL was adapted specifically for older adults and based on previously validated scales for the assessment of stressful life events: Social Readjustment Rating Scale [35], Recent Life Changes Questionnaire [36], and Life Events and Difficulties Schedule [37]. Participants were asked if they had experienced the “death of a significant other” within the last 12 months (yes/no), and if so, who died.

The enabling variables social network/social support were measured using the 6-item Lubben Social Network Scale (LSNS-6) [38]. The LSNS-6 consists of 6 questions assessing the size of the respondent’s active social network (ie, number of relatives or friends seen or heard from ≥1 time per month), perceived social support (ie, number of relatives or friends who could be called for help), and perceived confidant network (ie, number of relatives or friends with whom the respondent could talk about private matters). Each LSNS-6 question is scored on a 0-5 scale. The total social network score is an equally weighted sum of these 6 questions, with scores ranging from 0 to 30. Higher scores indicate larger social networks or more frequent social contact. It has been demonstrated that the scale has good internal consistency (Cronbach α=.83) [38].

### Statistical Analyses

Based on population statistics from the German Federal Statistics Office [39], data were weighted by USUMA in accordance with age, gender, and region. In order to obtain population-representative results, USUMA used design and adjustment weighting techniques [40]. In this study, unweighted absolute frequencies were presented, whereas any other analyses were performed and reported by using the weighting factor.

Statistical analyses were conducted using Statistical Package for the Social Sciences (SPSS) version 24.0 for Windows (SPSS Inc.). In the analysis, the variable frequency of internet use was recoded into a dichotomous variable: *yes* and *no*. Individuals who reported never using the internet were classified as noninternet users. Individuals who reported using it less than once a month or more were classified as internet users. In addition, the variable frequency of internet searches for physical or psychological health information was recoded into a binary variable *yes* and *no*. Further, marital status was recoded into a binary variable (single vs married/with partner), and a new dichotomous variable *living situation* (living alone/living with others) was created. According to the international education classification “CASMIN” [41], the variable educational level was created with 3 categories (low, middle, and high). Finally, the variable “chronic diseases” was computed by counting the number of chronic conditions. This criterion was met if 75% or more of the 22 items on the list of chronic conditions were checked positive. If there were more missing values on the list (>15 unanswered items), the question was considered invalid (N=1).

Descriptive statistics presented are means and corresponding SDs, absolute frequencies, and percentages, as appropriate. The 2 groups (internet users/nonusers) were compared using independent samples *t* tests (unpaired) or chi-square tests as appropriate. Next, binary logistic regression analyses were conducted to examine factors associated with internet use in older adults. The dichotomized variable internet use served as the dependent variable. The predictor variables included age, gender, education, marital status, domicile, experiences of social loss, GDS score, LSNS score, loneliness score, chronic disease, and EQ-5D-5L-VAS score. Furthermore, we performed a binary logistic regression analyses to investigate if health-related conditions are associated with internet search for physical or psychological health information within older internet users. The dichotomized variable internet search for physical or psychological health information (yes/no) served as the dependent variable. The predictor variables included GDS score and number of chronic disease. The significance level was set at *P*≤.05 for all analyses.

## Results

### Participant Characteristics

Sociodemographic characteristics of the study sample and comparison between internet users and nonusers are presented in Table 1. The sample consisted of 999 individuals; 612 (59.1%) were female and 387 (40.9%) were male. The average age was 80.49, with a range of 75-99 years. More than one-third of the respondents had a low educational level (368/999, 36.3%), roughly one-third was highly educated (324/999, 31.3%), and 32.4% (307/999) had a middle educational level.

**Table 1:** Sociodemographic characteristics of the sample (N=999).

Variables			All		Internet use				*P*-value
			(N=999, 100%)		Yes (n=418, 41.8%)		No (n=581, 58.2%)		
**Age (years)**									<.001
	Mean (SD)	80.49 (4.69)		78.91 (3.57)		81.67 (5.07)			
	Range	75-99		75-92		75-99			
**Gender, n^a^ (%^b^)**									<.001
	Male	387 (40.9)		231 (57.5)		156 (26.6)			
	Female	612 (59.1)		187 (42.5)		425 (73.4)			
**Education^c^, n (%)**									<.001
	Low	368 (36.3)		74 (17.7)		294 (50.1)			
	Middle	324 (32.4)		142 (33.6)		182 (31.5)			
	High	307 (31.3)		202 (48.7)		105 (18.4)			
**Domicile^d^, n (%)**									<.001
	Alone	572 (55.4)		185 (43.3)		387 (65.6)			
	With spouse	378 (38.5)		218 (53.1)		160 (28.4)			
	With relatives	46 (4.5)		17 (4.1)		29 (4.9)			
	Other	14 (1.5)		4 (0.9)		10 (1.0)			
	Missing	1 (0.1)		0 (0)		1 (0.1)			
**Marital status, n (%)**									<.001
	Married, living together	353 (36.3)		203 (49.3)		150 (26.6)			
	Married, living apart	25 (2.6)		15 (3.8)		10 (1.8)			
	Single	74 (7.5)		26 (6.4)		48 (8.3)			
	Divorced	77 (7.6)		28 (6.4)		49 (8.5)			
	Widowed	469 (46.0)		145 (34.0)		324 (55.8)			
	Missing	1 (0.1)		1 (0.1)		0 (0)			

^a^n: frequencies (none weight).

^b^%: percentages (weight).

^c^Educational classification according to the new CASMIN educational classification. Low: inadequately completed general education, general elementary education, basic vocational qualification or general elementary education, and vocational qualification; Middle: intermediate vocational qualification or intermediate general qualification and vocational qualification, intermediate general qualification, general maturity certificate, vocational maturity certificate/general maturity certificate, and vocational qualification; High: lower tertiary education—general diplomas/diplomas with vocational emphasis, higher tertiary education—lower level/higher level [41].

^d^Multiple responses possible.

### Differences Between Internet Use and Nonuse

**Figure 1:**
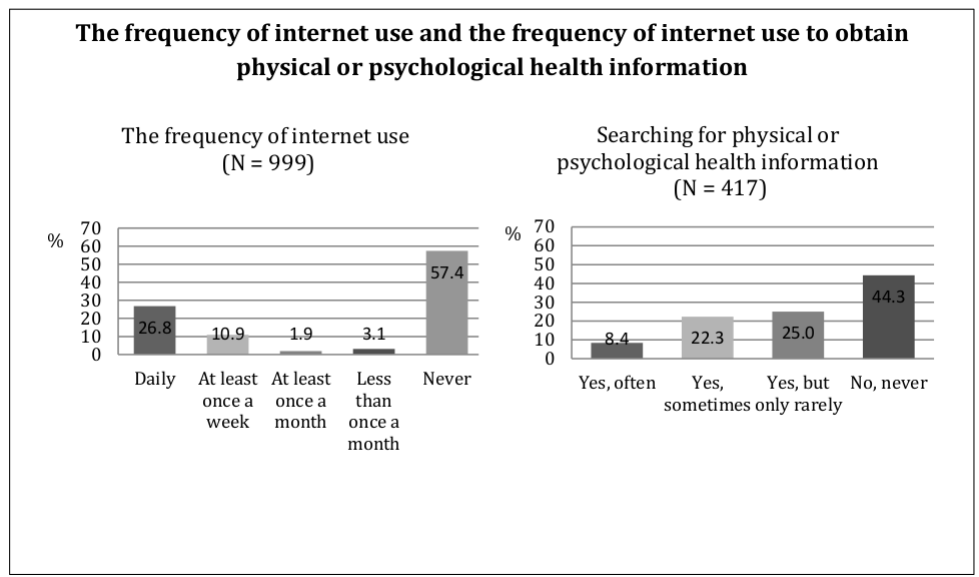
Frequency of internet use and frequency of internet use to obtain physical or psychological health information.

The majority of the sample consisted of internet nonusers (581/999, 57.4% vs 418/999, 42.6%, internet users). Of those using the internet, more than one-half searched the internet for physical or psychological health information (233/417, 55.7%). Figure 1 displays the frequency of internet use in general, as well as the frequency of internet use to obtain health-related information.

On a group level, there were several differences between users and nonusers, which are shown in Table 1 for sociodemographic variables and in Table 2 for social and health-related factors. In comparison to nonusers, older adults using the internet were more likely to be male (*P*<.001), tended to be younger (*P*<.001), were more likely to be middle or highly educated (*P*<.001), and were more likely to be married/with partner (*P*<.001) as well as to be living with their partner in the same household (*P*<.001). With regard to social and health-related factors, individuals using the internet tended to have better self-rated health-related quality of life (*P*<.001), reported fewer depressive symptoms (*P*<.001), fewer chronic medical conditions (*P*=.02), and less loneliness (*P*<.001). Furthermore, internet users reported having a greater and more supportive social network than nonusers (*P*<.001). No significant differences were found regarding experiences of social loss (*P*=.87).

**Table 2:** Social and health outcomes of the sample (N=999).

Variables		All	Internet use			*P*-value	
		(N=999, 100%)	Yes (n=418, 41.8%)	No (n=581, 58.2%)			
**Health-related quality of Life VAS EQ-5D-5L^a^**					<.001		
	Mean (SD)	73.46 (19.51)	78.02 (16.48)	70.08 (20.87)			
	Range	0-100	10-100	0-100			
	Missing, n^b^ (%^c^)	4 (0.4)	1 (0.3)	3 (0.4)			
**UCLA Loneliness Scale^d^**					<.001		
	Mean (SD)	3.57 (1.02)	3.41 (0.80)	3.68 (1.15)			
	Range	3-9	3-9	3-9			
	Missing, n (%)	15 (1.6)	4 (0.8)	11 (2.1)			
**Experience of social loss**					.87		
	Yes, n (%)	297 (30.0)	126 (30.2)	171 (29.8)			
	No, n (%)	702 (70.0)	292 (69.8)	410 (70.2)			
**GDS^e^**					<.001		
	Mean (SD)	1.81 (2.07)	1.27 (1.58)	2.21 (2.30)			
	Range	0-13	0-12	0-13			
	Missing, n (%)	10 (1.0)	5 (1.0)	5 (0.9)			
**LSNS^f^**					<.001		
	Mean (SD)	15.90 (5.97)	17.57 (5.52)	14.64 (5.99)			
	Range	0-30	0-30	1-30			
	Missing, n (%)	39 (4.0)	12 (2.9)	27 (4.8)			
**Chronic diseases^g^**					.02		
	Mean (SD)	3.58 (2.33)	3.37 (2.13)	3.73 (2.46)			
	Range	0-13	0-12	0-13			
	Missing, n (%)	1 (0.1)	0 (0)	1 (0.2)			

^a^VAS EQ-5D-5L: Visual Analogue Scale of the EuroQol 5-Dimensions 5-Level Questionnaire; score ranges from 0 (worst imaginable health state) to 100 (best imaginable health state).

^b^n: frequencies (none weight).

^c^%: percentages (weight).

^d^UCLA: University of California, Los Angeles, Loneliness Scale; score ranges from 1 (hardly ever) to 3 (often).

^e^GDS: Geriatric Depression Scale; score ranges from 0 (no depression symptoms) to 15 (depression symptoms), cut-off: > 4.

^f^LSNS: Lubben Social Network Scale; score ranges from 0 to 30, with higher values reflecting more social networks and more social support.

^g^The sum score of chronic diseases ranges from 0 (no chronic conditions) to 22 (22 chronic conditions).

### Predictors of Internet Use

A binary logistic regression was conducted to identify associations between several factors and internet use (Table 3). Results revealed that younger individuals had significantly higher odds for internet use than older individuals (odds ratio [OR] 0.89, 95% CI 0.85-0.92; *P*<.001). In addition, gender was associated with internet use in older age: men were 2.8 times more likely to use internet than women. Furthermore, a higher educational level was significantly associated with internet use among older adults (OR 6.69, 95% CI 4.48-9.99; *P*<.001). Individuals who reported having a greater social network showed a higher chance for internet use than individuals with a smaller social network (OR 1.04, 95% CI 1.01-1.07; *P*=.01). Moreover, individuals with fewer depressive symptoms were 0.9 times more likely to use internet than those with more depressive symptoms. Participants with a better self-reported health-related quality of life had significantly higher odds for internet use than participants with lower self-reported health-related quality of life (OR 1.02, 95% CI 1.00-1.03; *P*=.006). Finally, the number of self-reported chronic diseases was associated with a higher likelihood of internet use among older adults: individuals who had a greater number of chronic diseases had 1.1 times the odds of reporting internet use than individuals with fewer chronic diseases, adjusting for all other sociodemographic and health-related covariates. No significant associations between experience of loneliness, experience of social loss, marital status, and domicile and internet use were found. In total, this model explained between 29.3% (Cox and Snell) and 39.3% (Nagelkerke *R*^2^) of the variance in internet use, and substantially improved model predictive power (from 56.8% to 73.8%).

**Table 3:** Results of binary logistic regression analysis for variables predicting internet use versus nonuse in older adults (N=937).

			Odds ratio (95% CI)	*P*-value	Wald
Age			0.888 (0.854-0.924)	<.001^h^	34.238
**Gender**					
		Male	2.842 (2.017-4.002)	<.001^h^	35.711
		Female	Reference		
**Education^a^**					
		Low	Reference		
		Middle	3.813 (2.582-5.630)	<.001^h^	45.295
		High	6.691 (4.479-9.995)	<.001^h^	86.146
**Marital status^b^**					
		Single	Reference		
		Married, living in partnership	1.136 (0.614-2.100)	.685	0.165
**Domicile**					
		Alone	Reference		
		Living with others	1.354 (0.741-2.473)	.324	0.973
GDS^c^			0.888 (0.795-0.992)	.036^i^	4.407
LSNS^d^			1.037 (1.008-1.067)	.014^i^	6.101
Health-related quality of Life VAS EQ-5D-5L^e^			1.015 (1.004-1.025)	.006^j^	7.526
Chronic diseases^f^			1.122 (1.037-1.213)	.004^j^	8.301
UCLA Loneliness Scale^g^			0.905 (0.748-1.094)	.303	1.062
**Experience of social loss**					
	Yes		0.855 (0.604-1.212)	.379	0.773
	No		Reference		
Constant			407.376	.001	11.855
Nagelkerke *R*^2^			0.393		
Log-likelihood			953.612		

^a^Educational classification according to the new CASMIN educational classification. Low: inadequately completed general education, general elementary education, basic vocational qualification or general elementary education, and vocational qualification; Middle: intermediate vocational qualification or intermediate general qualification and vocational qualification, intermediate general qualification, general maturity certificate, vocational maturity certificate/general maturity certificate, and vocational qualification; High: lower tertiary education-general diplomas/diplomas with vocational emphasis, higher tertiary education—lower level/higher level [41].

^b^Single: single, divorced, widowed; Married/living in partnership: married/with spouse, married/living apart).

^c^GDS: Geriatric Depression Scale; score ranges from 0 (no depression symptoms) to 15 (depression symptoms), cutoff: >4.

^d^LSNS: Lubben Social Network Scale; score ranges from 0 to 30, with higher values reflecting more social networks and more social support.

^e^VAS EQ-5D-5L: Visual Analogue Scale of the EuroQol 5-Dimensions 5-Level Questionnaire; score ranges from 0 (worst imaginable health state) to 100 (best imaginable health state).

^f^The sum score of chronic diseases ranges from 0 (no chronic conditions) to 22 (22 chronic conditions).

^g^UCLA: University of California, Los Angeles, Loneliness Scale; score ranges from 1 (hardly ever) to 3 (often).

^h^Significant on the level α=.001.

^i^Significant on the level α=.05.

^j^Significant on the level α=.01.

In order to test the association between health-related factors and the internet use for health purposes among internet users a binary logistic regression was conducted. Neither depressive symptoms (OR 1.10, 95% CI 0.962-1.260, *P*=.164) nor the number of chronic diseases (OR 1.03, 95% CI 0.932-1.132, *P*=.586) predicted internet search for health purposes.

## Discussion

### Principal Findings

The aim of this study was to investigate frequency of and factors associated with internet use among individuals aged 75 plus in Germany. Our results revealed that almost half of the 75- to 99-year old reported some internet use. This is comparable to the rates for adults aged 70 plus reported by a population survey on general internet use in Germany in 2016 [17]. Taking into account the trend of internet usage among this age group, there is evidence of a rapid increase of internet users in Germany [17]. Frees and Koch [17] showed that internet usage among this age group is on the rise (up to 65% in 2018), noting that older adults have the highest increase of daily internet use (from 25% in 2016 to about 40% in 2018). Our results confirm these findings—26.8% (261/999) of the 75-99-year olds used the internet daily. At the same time, we expanded existing data by examining factors related to internet use in older and oldest age. In sum, regression analyses showed that male gender, age, higher levels of education, a more extensive social network, higher level of health-related quality of life, fewer depressive symptoms, and a higher number of chronic diseases increased the likelihood of internet use in this age group.

### Possible Explanations and Relation to Previous Studies

As for predisposing characteristics, the few studies that have focused on older adults (75+) [12,42-44] have shown that internet use in this age is associated with male gender, higher levels of education, being married, and reporting a better health status. In accordance therewith, our findings demonstrate that demographic and socioeconomic status variables are indeed significant predictors of internet use among this age group in Germany. Two of the strongest predictors are male gender and higher levels of education. van Deursen et al [45] showed in their (older adults) sample of internet users and nonusers in the Netherlands that women tend to not make use of the existing internet connection at home. The authors concluded that internet use among older adults seems to be a male-dominated activity. This may be due to the notion that ICT-related skills have been historically stereotypically perceived as more masculine (eg, males are good with technology). Among the older and oldest, such traditional stereotypes may play a more prominent role. However, as Baby Boomers get older, this may change [46], as younger generations of women are more familiar with using the internet and ICT (eg, smartphones).

As for the enabling factor social network/social support, previous studies that examined determinants of internet use among older adults have found that internet users also differ from nonusers with regard to their social embeddedness. Our findings are in accordance with previous research showing that internet users with a more extensive social network are more likely to use the internet [47]. However, due to the correlational nature of this study, no assertion can be made about causality. Therefore, the use of the internet might increase users’ social network as measured in this study. Thus, there are other studies which highlight the potential of internet use to enhance or maintain social networks [2] and reduce feelings of loneliness [48] and social isolation [3]. Despite this, and in line with previous studies [12,45], our study found that support from partners, family members, or friends seems to effect the likelihood of going online.

As for need factors, existing studies show associations between internet use and physical/mental health and well-being [2,8,10,14] in this age group. Our findings also reflect this and show that the self-reported health-related quality of life increases the odds for internet use. In addition, a higher number of chronic medical conditions increased the likelihood of internet use in our multivariate analysis. A possible explanation might be the increased need for information about the disease and treatment options. Dumitru et al [19], for instance, noticed that using the internet for health-related purposes is very common (53.7%) and quickly increasing. Accordingly our findings show that more than half of the interviewees using the internet search on it for physical or psychological health information. Furthermore, findings from several studies show high rates of health-related internet use among those with multiple medical conditions or long-term illness [15,19]. However, this contrasts with our finding (ie, no link between internet use for health-related purposes and health-related factors such as the number of chronic medical conditions as well as depressive symptoms). Age seems to be negatively associated with health-related internet use [19,49,50]. This may be due to the fact that old and very old people with multiple medical conditions in Germany prefer other sources of information about health or illness, for example, direct face-to-face contacts with physicians and other health care professionals [19].

Common critical life events in older age are social loss experiences along with grief and bereavement, which are considered as risk factors for the development of mental health problems, especially depression and loneliness [51-53]. Whereas individuals with fewer depressive symptoms are more likely to use the internet in this study, no statistically significant effect of experiences of social loss or loneliness on an intensified use of internet among older adults was found. However, this does not necessarily mean older people would not benefit from ICT, for example, internet-based mental health interventions. Research has shown that eHealth (electronic health) interventions may significantly reduce mental health problems in older adults (eg, anxiety and depression [54-56]). Further research targeting this issue is currently being conducted [57].

### Strengths and Limitations

The main strength of this study is the gathering of data from a population-based sample of the general German population aged 75 years and older that can be generalized to other populations in old age. Representativeness was ensured by using design and adjustment weighting methods of the data according to age, gender, and region [40] based on population statistics from German Federal Statistics [46]. However, it should be noted that representativeness may be limited due to the reduction of the sample size for analysis and because only community-dwelling older adults with landline telephones, adequate hearing and speech comprehension, and without cognitive impairment were included in the sample. Apart from this, the study has further limitations. First, as the data from the telephone survey offer only a cross-sectional data set, no conclusion about long-term effects and causality can be drawn. Second, we aimed to incorporate many factors that may influence internet use, but, of course, it is likely that there were other unobserved variables at play, for instance, previous internet experiences or internet skills are associated with preferences in internet use in the older age group. Third, the survey is based on self-reported measures; therefore influences such as social desirability cannot be excluded. For more in-depth understanding of the use or nonuse of the internet and the underlying purposes qualitative research might be useful.

### Conclusions

Today, public and private sectors commonly offer online services, which, in turn, have an influence on economic, cultural, and private life. ICT facilities include every day activities such as emailing, online banking, and information seeking, as well as increasingly eHealth services. For example, support via the internet, internet-based therapeutic interventions, and assistive technology are gaining in popularity. Hence, internet-based technologies have become a bigger part of the lives of older adults [58]. Therefore, there is a growing number of studies in this research field focusing specifically on older adults. The studies have identified factors associated with internet usage, as well as benefits and barriers. Moreover, eHealth interventions seem to be promising for promoting older people’s well-being by fostering active aging or to helping the elderly stay independent as long as possible. Most of the current research has been mainly focused on younger people or has been limited to pilot studies [59]. Therefore, further research is needed, especially research identifying the types of older adults who would benefit the most from ICT use.

## Appendix

Multimedia Appendix 1 Study sampling flowchart.

## Abbreviations

AMD: Association for German Market and Social Research Agency
CASMIN: CASMIN Educational Classification in International Comparative Research
DFG: German Research Foundation
GDS: Geriatric Depression Scale
ICT: Information and Communication Technology
LLL: Leipziger Lebensereignis-Liste
LSNS: Lubben Social Network Scale
SPSS: Statistical Package for the Social Sciences
UCLA: University of California, Los Angeles, Loneliness Scale
