# Supplementary figures and images for: Internet Use in Old Age: Results of a German Population-Representative Survey
Source: J Med Internet Res. 2020 Nov 23;22(11):e15543. doi: 10.2196/15543 (PMC7685698; doi:10.2196/15543)

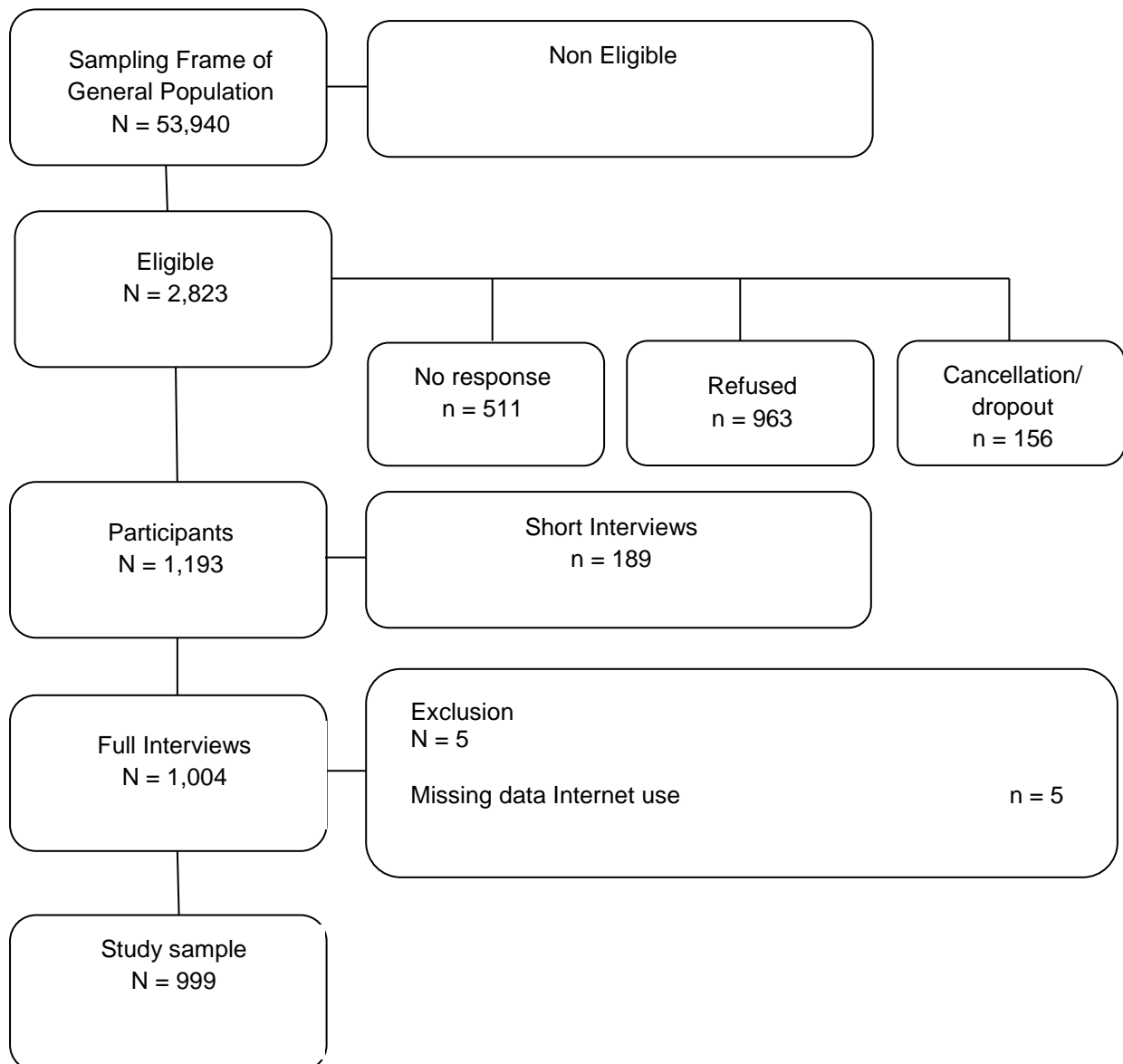

Supplement: Multimedia Appendix 1 [file jmir_v22i11e15543_app1.pdf]
